# Non-invasive maturity assessment of iPSC-CMs based on optical maturity characteristics using interpretable AI

**DOI:** 10.1016/j.csbj.2025.08.024

**Published:** 2025-08-22

**Authors:** Fabian Scheurer, Alexander Hammer, Mario Schubert, Robert-Patrick Steiner, Oliver Gamm, Kaomei Guan, Frank Sonntag, Hagen Malberg, Martin Schmidt

**Affiliations:** a Institute of Biomedical Engineering, TU Dresden, Fetscherstr. 29, Dresden 01307, Germany; b Fraunhofer Institute for Material and Beam Technology IWS, Winterbergstr. 28, Dresden 01277, Germany; c Institute of Pharmacology and Toxicology, TU Dresden, Fetscherstr. 74, Dresden 01307, Germany

**Keywords:** Maturity assessment, IPSC-CM, Video-based motion analysis, Optical characteristics, Interpretable AI, Machine Learning, Non-invasive

## Abstract

Human induced pluripotent stem cell-derived cardiomyocytes (iPSC-CMs) are an important resource for identifying novel therapeutic targets and cardioprotective drugs. However, a key limitation of iPSC-CMs is their immature, fetal-like phenotype. Cultivation of iPSC-CMs in lipid-supplemented maturation media (MM) enhances the structural, metabolic and electrophysiological properties of iPSC-CMs. Nevertheless, they face substantial limitations as there are labor-intensive, time consuming and go in line with cell damage or loss of the sample. To address this issue, we have developed a non-invasive approach for automated classification of iPSC-CM maturity through interpretable artificial intelligence (AI)-based analysis of beat characteristics derived from video-based motion analysis. In a prospective study, we evaluated 230 video recordings of early-state, immature iPSC-CMs on day 21 after differentiation (d21) and more mature iPSC-CMs cultured in MM (d42, MM). For each recording, 10 features were extracted using Maia motion analysis software and entered into a support vector machine (SVM). The hyperparameters of the SVM were optimized in a grid search on 80 % of the data using 5-fold cross-validation. The optimized model achieved an accuracy of 99.5 ± 1.1 % on a hold-out test set. Shapley Additive Explanations (SHAP) identified displacement, relaxation-rise time and beating duration as the most relevant features for assessing iPSC-CM maturity. Our results suggest the use of non-invasive, optical motion analysis combined with AI-based methods as a tool to assess iPSC-CMs maturity and could be applied before performing functional readouts or drug testing. This may potentially reduce the variability and improve the reproducibility of experimental studies.

## Highlights

• Non-invasive maturation state evaluation of human iPSC-CMs by analyzing beating characteristics using interpretable AI.
• Distinguish immature from more mature iPSC-CMs with high accuracy of 99.5 % using a simple support vector machine.
• Methods of explainable artificial intelligence enable the identification of the most relevant beating characteristics .
• Optical evaluation of iPSC-CM maturation state may reduce experimental variability and improve the reproducibility of studies.

## Graphical Abstract

**Figure:**
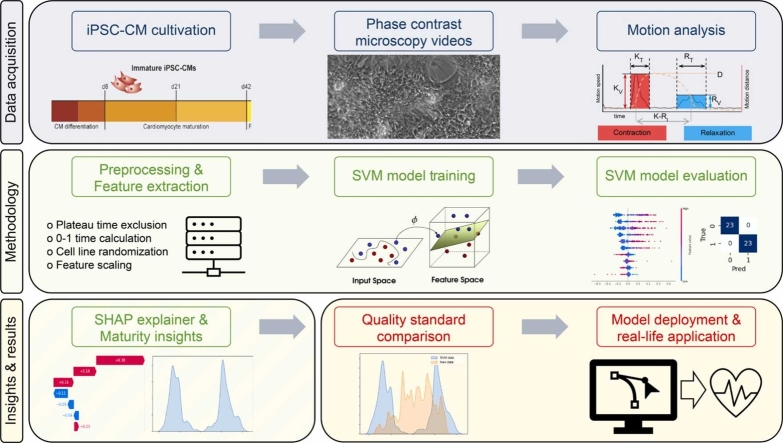

## 1 Introduction

The development of human induced pluripotent stem cell-derived cardiomyocytes (iPSC-CM) has the potential to advance both basic research and clinical applications [1], [2]. However, the immature phenotype of iPSC-CMs in comparison to adult CMs represents a major limitation for their utilization in drug screenings or the recapitulation of clinical disease phenotypes in vitro [3], [4], [5], [6].

Different methods have been described to enhance the structural, metabolic and functional properties of iPSC-CMs, including lipid-supplementation of culture media, patterning of the culture surface, optimized extracellular matrix composition, electrostimulation, or cultivation in 3D tissue models with other cardiac cell types (reviewed in [7]). Furthermore, recent studies highlight the relevance of the maturation state of iPSC-CMs for their sensitivity towards pathophysiological stimuli, such as hypoxia-induced cell death [6], [8], and their response to cardioactive drugs [9], [10], [11]. In particular, an enhanced maturation of iPSC-CMs was shown to improve their potential to detect pro-arrhythmic effects and predict the safety margin of cardioactive drugs [10]. Consequently, assessing the maturation state of iPSC-CMs is important for data interpretation and comparison between different experiments or iPSC lines.

The maturation state of iPSC-CMs is usually evaluated based on analyzing structural, electrophysiological, functional, or metabolic cell properties as well as protein- and gene expression [12]. Most of the experimental techniques to assess iPSC-CM maturity are time-consuming, costly, and destructive, as they require harvesting, fixation, replating, or the application of fluorescent dyes with long-term toxicity [13].

In contrast, the spontaneous beating activity of iPSC-CMs represents a functional hallmark of iPSC-CMs, which can be flexibly assessed during long-term culture and quantified via video-based motion analysis [14], [15], [16]. Using this technique, a broad set of different features describing the contractile activity can be obtained, including beating rate, contraction and relaxation time (C time and R time), maximum contraction and relaxation velocity (Max C and Max R), and beating duration (0–1 time). Importantly, recently published studies revealed changes in these beating features during maturation of iPSC-CMs [16], [17], [18].

We therefore hypothesized that analyzing the spontaneous beating activity of iPSC-CMs provides a non-invasive approach to assess their maturation state.

Artificial intelligence (AI), including machine learning or deep learning, offers great potential for interpretation of data from cell-based studies and has been used for drug cardiotoxicity testing [19] or the discrimination of atrial and ventricular cells [16], but not for the assessment of iPSC-CM maturity. A previous study demonstrated the application of supervised machine learning on multidimensional data from iPSC-CMs and engineered cardiac tissues subjected to drug exposure, enabling automated determination of cardioactive drugs and prediction of their mechanism of action [20].

Due to the lack of gold standards or necessary data, the use of AI is often directed toward simpler machine learning models. Support vector machines (SVMs) were commonly applied as learning models with remarkable results [21], [22], [23], [24]. These models offer high generalizability, can be applied to smaller data sets, require few computational resources and are suitable for explanation models regarding the feature importance compared to more complex deep learning models. The interpretability of processed features is a key advantage of SVMs.

**Fig. 1:**
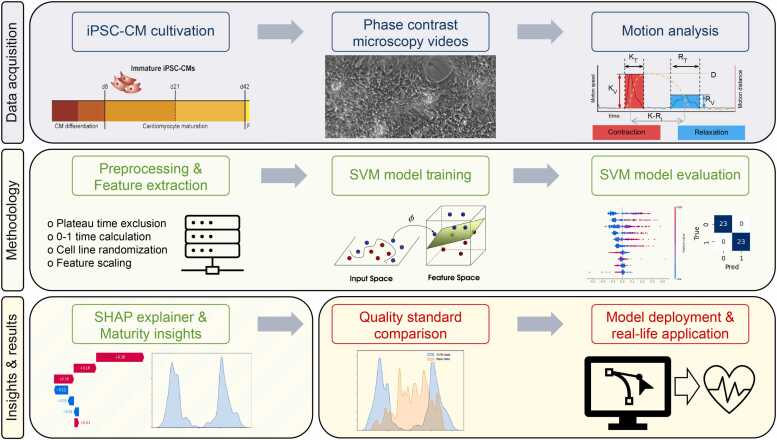
Overview of iPSC-CM culture, data acquisition, model training for maturity classification, and analysis of individual parameter’s influence on maturity classification. SHAP, Shapley additive explanations; SVM, support vector machine; iPSC-CM induced pluripotent stem cell-derived cardiomyocytes.

The objective of this study was to investigate the potential of interpretable AI, utilizing SVMs and explanation models, in differentiating between mature and immature iPSC-CMs based on the analysis of their beating features (Fig. 1). Furthermore, we aimed to investigate which beating parameter or combination of beating features is most suitable for estimating iPSC-CM maturation.

## 2 Material and methods

### 2.1 Data collection and iPSC-CM cultivation

In this study, we used data of iPSC-lines generated from 4 healthy donors. iBM76 (UMGi005-A) and iWTD2 (UMGi001-A) cells were reprogrammed from mesenchymal stem cells and dermal fibroblasts, respectively, using STEMCCA lentivirus, and were characterized previously [25], [26]. Cell lines isWT7 and isWT1 were reprogrammed from dermal fibroblasts using the integration-free CytoTune-iPS 2.0 Sendai Reprogramming Kit (Thermo Fisher Scientific) and characterized as reported recently [11]. The generation of iPSCs was approved by the Ethics Committee of the University Medical Center Göttingen (approval number: 21/1/11 and 10/9/15) and performed according to the approval guidelines.

Directed cardiac differentiation was performed by modulation of Wnt signaling as previously described [27], [28]. Briefly, iPSCs were grown to 85–85 % confluency on Geltrex-coated (Thermo Fisher Scientific) plates in Essential 8 medium (E8 medium, Thermo Fisher Scientific) with daily medium changes. Differentiation of iPSCs was performed using cardiac differentiation medium (C-diff), consisting of RPMI 1640 with Glutamax and HEPES (Thermo Fisher Scientific), supplemented with 0.2 mg/ml ascorbic acid 2-phosphate (Sigma-Aldrich) and 0.5 mg/ml human recombinant albumin (Sigma-Aldrich). To initiate cardiac differentiation (day 0), E8 medium was replaced with C-diff supplemented with 4 µM CHIR99021 (Merck Millipore). After 48 h, the medium was exchanged for C-diff containing 5 µM IWP-2 (Merck Millipore) for an additional 48 h. On days 4 and 6, the medium was exchanged for C-diff, and from day 8 onwards, cells were cultured in RPMI 1640 with 2 % B27-supplement (B27 medium, Thermo Fisher Scientific). Afterwards, medium was changed every other day. First contractions were observed between days 8–10. Between days 14–16, iPSC-CMs were replated into Geltrex-coated 6-well plates. Therefore, the medium was aspirated, and the cells were incubated in RPMI 1640 with 1 mg/ml collagenase B (Worthington) for 30–60 min. Detached iPSC-CM sheets were transferred into 0.25 % trypsin/EDTA (Thermo Fisher Scientific) and incubated for 8 min at 37°C. Cells were resuspended in RPMI 1640 with 20 % FBS and 2 µM thiazovivin (Merck Millipore), counted using a hemocytometer (Neubauer improved), and seeded into Geltrex-coated 6-well plates at a density of 0.5 million cells/well. After 24 h, the medium was replaced with B27 medium. On day 21, iPSC-CMs were randomly assessed into 2 experimental groups cultured in either B27 medium or maturation medium (MM). MM consisted of DMEM without glucose (Thermo Fisher Scientific), 7 mM glucose (Sigma-Aldrich), 0.8 mM lactate (Sigma-Aldrich), 1.6 mM L-carnitine-hydrochloride (Sigma-Aldrich), 5 mM creatine-monohydrate (Sigma-Aldrich), 2 mM taurine (Sigma-Aldrich), 0.5 mM L-ascorbic acid-2-phosphate (Sigma-Aldrich), 0.5 % AlbuMax I lipid-rich BSA species (Thermo Fisher Scientific), 2 % B27-supplement without insulin (Thermo Fisher Scientific), 50 nM human insulin (Sigma), 1 % Knockout Serum replacement (Gibco), 1 % non-essential amino acids (Gibco), 82 nM biotin (Sigma-Aldrich), and 0.37 nM vitamin B12 (Sigma-Aldrich). The culture medium was replaced every other day (2 ml per well).

### 2.2 Video acquisition and motion analysis

**Fig. 2:**
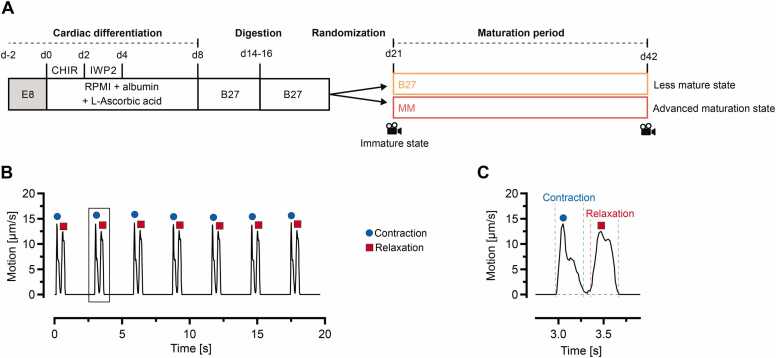
Experimental design, data acquisition and analysis. A, Scheme of experimental groups and video recording time points (d21 and d42). B, Representative motion trace of iPSC-CMs obtained using Maia software. C, Magnified view of the region of interest highlighted in B, showing a single beating cycle consisting of contraction and relaxation phase.

The spontaneous beating activity of iPSC-CM was recorded at different time points to assess iPSC-CMs at distinct maturation states (Fig. 2A). To evaluate the beating status of immature iPSC-CMs, video recordings were performed on day 21, prior to distribution into experimental groups receiving either B27 medium or MM. Video data from iPSC-CMs at day 42, after 21 days in MM, were used to define the beating properties of more mature iPSC-CMs, was as previously characterized by molecular and functional analyses [11].

For improved readability, iPSC-CMs on day 21 were designated as ‘immature’, while iPSC-CMs cultured in MM were designated as ‘mature’ on day 42, recognizing that functional maturity may not be fully achieved under all conditions.

Two videos were recorded for each well/culture at different, randomly selected positions. Videos were captured in phase contrast mode using a Hamamatsu ORCA Flash 4.0 V3 CMOS camera (Hamamatsu Photonics) at a length of 20–30 s, a frame rate of 60 Hz and a resolution of 0.65 µm/pixel in an area of 670 × 670 µm (1024 × 1024 pixel). Recordings were performed using ZEN software (Carl Zeiss) and analyzed using the Maia software [28]. Maia detects iPSC-CM motion using block-matching, similar to OpenHeartWare [14] and MUSCLEMOTION [29]. Block width (16 pixel, which corresponds to 10.4 µm) and maximum shift (7 pixel, which corresponds to 4.55 µm) were set according to Huebsch et al. [14]. Frame offset was manually determined to 100 ms (4 frames offset), as this provided most stable detection of max. relaxation velocity (Max R) and relaxation time (R time). Examination of the influence of different brightness levels on the individual beating features revealed that C-rise time, and to a lesser extent beating duration decreased with increasing brightness (Supplementary Figure 1). Importantly, the Maia software allows manual adjustment of the brightness after loading the video. Contraction and relaxation peaks as well as motion start and endpoints were manually adjusted.

### 2.3 Feature extraction

**Fig. 3:**
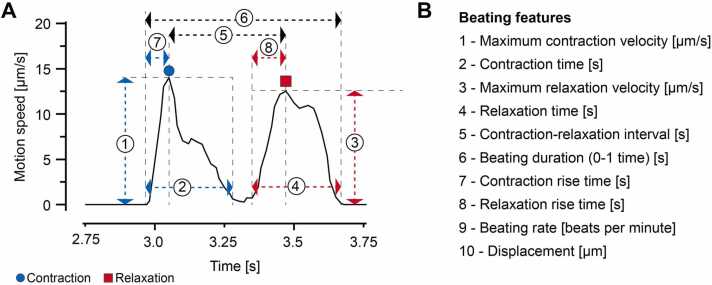
Extracted features. Representative beating cycle (A) and list of extracted beating features (B). Displacement was calculated as the integral of the movement speed during the contraction phase. Maxima as well as start and end points of contraction and relaxation were manually adjusted.

For each video, 10 features were extracted using the Maia (Fig. 3). The feature time series, i.e. the representation of features over time, were z-normalized feature-wise to counteract strong differences in parameter expressions.

Due to the optimization of Maia during its development, in 33.1 % of all samples, the 0–1 time was missing. Accordingly, the 0–1 time was calculated by adding together the contraction time, relaxation time and the mean plateau time for each cell line.

Data preparation and all following operations were performed using Jupyter Notebook running on a Python 3 kernel. The scikit-learn package [30] version 1.2.2 was used.

### 2.4 Data splits

The overall dataset contained 362 videos of which 130 videos derived from immature iPSC-CMs (day 20/21, from 10 independent differentiations of 4 iPSC lines), 115 videos derived from mature iPSC-CMs cultured in MM (day 42, MM from 14 independent differentiations of 4 iPSC lines), and 117 were obtained from iPSC-CMs with an intermediate maturation state cultured in B27 medium (day 42, B27, from 16 independent differentiations of 4 iPSC lines).

Given the experimental setup and video capturing, mature cells (n = 115 videos, MM at day 42) were less represented than immature cells (n = 130 videos, day 21). Furthermore, the different cell lines were not equally represented at each maturity level. Therefore, we picked n = 230 recordings pseudo-randomly to equalize the number of recordings per maturity level (115 recordings per maturity level), considering an equal distribution of the represented cell lines. The data was partitioned into a training/validation set and a test data set in the ratio of 80–20 as summarized in Table 1. Thus, 184 samples (92 videos each from iPSC-CMs at day 21 and in MM at day 42) were employed for training and validation in a 5-fold cross-validation, which was repeated multiple times in a grid search for hyperparameter optimization. The final model was tested on the 46 retained samples. The partitioning was pseudo-randomized (random seed 42) and stratified, considering balanced classes and homogeneously distributed cell lines.

**Table 1:** Data distribution per split regarding date of recording, day 21 (cultured in B27 medium) and day 42 (cultured in MM), and cell line.

**Data split**	**Day 21, B27**		**Day 42, MM**		**Total**	
	*n*	*Cell lines*	*n*	*Cell lines*	*n*	*Cell lines*
**Training/validation set** *5-fold cross-validation*	92	4	92	4	184	4
**Test set** *Hold-out*	23	4	23	4	46	4
**Total**	115		115		230	

### 2.5 Maturity classification model development

A SVM model separates two classes by calculating the offset and direction of a plane, in our application the mature and immature cell cultures being on opposite sites. It does so in maximizing a margin m, as part of the plane’s direction vector $\vec{w}$ around this hyperplane in an n-dimensional space. By introducing a slack variable $\xi_{i}$, the model can be further optimized to tolerate classification errors. The variable C controls the way misclassifications are tolerated according to Eq. (1) [31]. The minimization term $0.5∙{\left\|\vec{w}\right\|}^{2}$ is added with the product of the regularization variable C times the sum of the slack variables $\xi_{i}$ for every sample [30], [31], [32].

(1) $$\vec{w},t,\xi_{i}=\arg\underset{\vec{w},t,\xi_{i}\;}{\min}\frac{1}{2}∙{\left\|\vec{w}\right\|}^{2}+C∙\sum_{i=1}^{n}\xi_{i}$$

An instance of a SVM model was created. After fitting the model with the training set, it was analyzed using the confusion matrix and its derived evaluation functions from scikit-learn in Python.

For hyperparameter optimization, an initial random search was performed, followed by a detailed grid search around the hyperparameter combination resulting from random search. In the initial random search, the optimal precision was sought by altering hyperparameters over 200 iterations in a grid search. This included the regularization parameters *C* (reciprocal(0.1, 3)), different *kernel* functions (radial basis function, polynomial, sigmoid), the influence of a single training example *gamma* (auto, scale), the *degree* of the polynomial kernel (2, 3, 4), and the offset parameter *coef0* (uniform(0,1)). During this process, training and test parameters were recorded and evaluated. The parameters of the best-performing model were then used as the starting point for the subsequent grid search.

In the grid search, the model was optimized by varying 5 hyperparameters, including *C* (0.1: 0.1: 1), *kernel* (radial basis function, polynomial), *gamma* (auto, scale), *degree* (2, 3), and *coef0* (0: 0.05: 1). Model optimization was performed using classification accuracy as the evaluation metric, assessed through 5-fold cross-validation.

### 2.6 Model evaluation

Model accuracy evaluation: After the 5-fold cross-validation and hold-out testing, we performed another 5-fold cross-validation on the full dataset (n = 230) using the same hyperparameter combination. In addition to accuracy, precision, recall, and F1-score were averaged across all folds, respectively. To estimate the robustness of the model, we calculated the standard deviation (SD) of the metrics across all folds [33].

Evaluation of class separability: The underlying structure of SVMs as geometric models enables further analysis on the separability of classes in the feature space. We hypothesized that the distance from the hyperplane that separates the two classes may correlate with the maturity of a cell culture. Therefore, we examined the separability of both classes using the decision function in the scikit-learn package for Python [30].

Evaluation of feature relevance: We assessed the feature relevance for the automated classification decision and compared it with cell biological *a priori* knowledge. To explain the SVM model and quantify feature relevance values, we applied Shapley Additive Explanation (SHAP) [34]. SHAP is a model agnostic approach that quantifies the contribution $\phi_{\mathrm{val}}$ of each individual feature F to the classification decision. This provides an explanation of how the presence of the values i within the features in any parameter selection and composition $S\subseteq F\setminus \left\{i\right\}$, affects the model prediction. The average contribution of i from the selection S is normalized and summed up for all possible parameter combinations, as seen in Eq. (2) [34].

(2) $$\phi_{\mathrm{val}}\left(i\right)=\sum_{S\subseteq F\setminus \left\{i\right\}}\frac{\left|S\right|!\left(p-\left|S\right|-1\right)!}{p!}\Delta \mathrm{val}\left(i,S\right)$$

## 3 Results

**Fig. 4:**
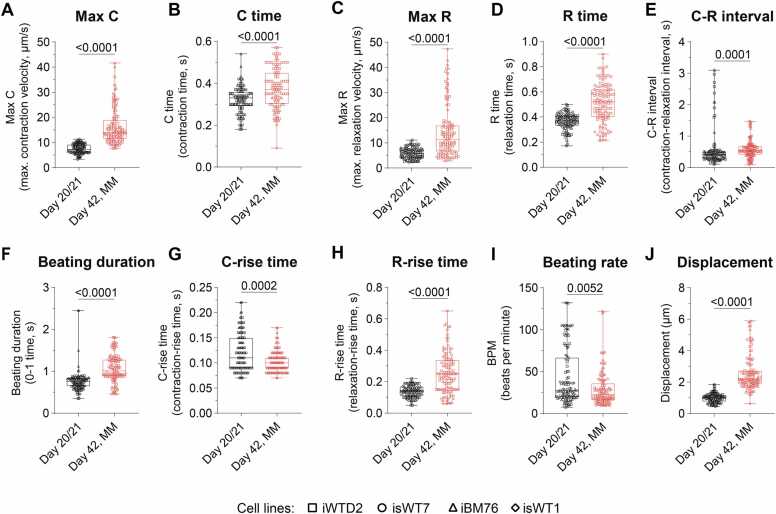
Beating characteristics of early-state, immature iPSC-CMs at day 21 and more mature iPSC-CMs after cultivation in MM at day 42. A-J; A, Maximum contraction velocity (Max C); B, Contraction time (C time); C, Maximum relaxation velocity (Max R); D, Relaxation time (R time); E, Contraction-relaxation interval (C-R interval); F, Beating duration; G, Contraction-rise time (C-rise time); H, Relaxation-rise time (R-rise time); I, Spontaneous beating rate; J, Displacement. Data from n = 230 videos obtained from 10 (day 21; n_21_ = 115) or 14 (day 42, n_42,MM_ = 115) independent differentiations of iPSC-CMs derived from iPSC-lines of 4 different donors (iWTD2, isWT7, iBM76, isWT1, indicated by symbols). Statistical analysis was performed using the Kruskal-Wallis test with Dunn’s correction for multiple comparisons.

Beating features extracted from video-based motion analysis were used to train a SVM model to discriminate between immature and mature iPSC-CMs. Cells cultured in MM were considered to represent a more mature state compared to those in B27 medium, based on previous reports demonstrating an enhanced electrophysiological function, calcium handling, metabolic activity [11]. Comparison of the beating features between both groups revealed strong differences between immature iPSC-CMs at day 20/21 and mature iPSC-CMs cultured in MM at day 42 (Fig. 4). Mature cells exhibited significantly higher Max C, Max R and displacement (p < 0.0001, Fig. 4A, C, J), prolonged C time, R time, C-R interval, beating duration, and R-rise time (p < 0.0001, Fig. 4B, D-F, H), shortened C-rise time (p = 0.0002, Fig. 4G) and a reduced spontaneous beating rate (p = 0.0052, Fig. 4I).

After hyperparameter optimization, the models scored on the test set with an accuracy of 99.5 ± 1.1 % (mean ± SD). The precision achieved on the test set was 100.0 ± 0.0 %, the recall was 98.8 ± 2.5 %, and the F1-score was 99.4 ± 1.3 %. The accuracy of the cross-validation on the full dataset was 99.1 ± 1.1 %, the precision was 99.1 ± 1.9 %, the recall was 98.8 ± 2.5 %, and the F1-Score was 99.1 ± 1.1 % (Table 2).

**Table 2:** Evaluation metrics results for test set, cross validation set and full data set on grid search model optimized for best accuracy. SD, standard deviation.

	***n***	**Accuracy** *mean ± SD*	**Precision** *mean ± SD*	**Recall** *mean ± SD*	**F1-Score** *mean ± SD*
**Validation set** *5-fold cross-validation*	184	99.3 ± 0.43 %	100.0 ± 0.0 %	98.6 ± 0.9 %	99.3 ± 0.5 %
**Test set** *hold-out*	46	99.5 ± 1.1 %	100.0 ± 0.0 %	98.8 ± 2.5 %	99.4 ± 1.3 %
**Full data set** *5-fold cross-validation*	230	99.1 ± 1.1 %	99.1 ± 1.9 %	99.2 ± 1.5 %	99.1 ± 1.1 %

**Fig. 5:**
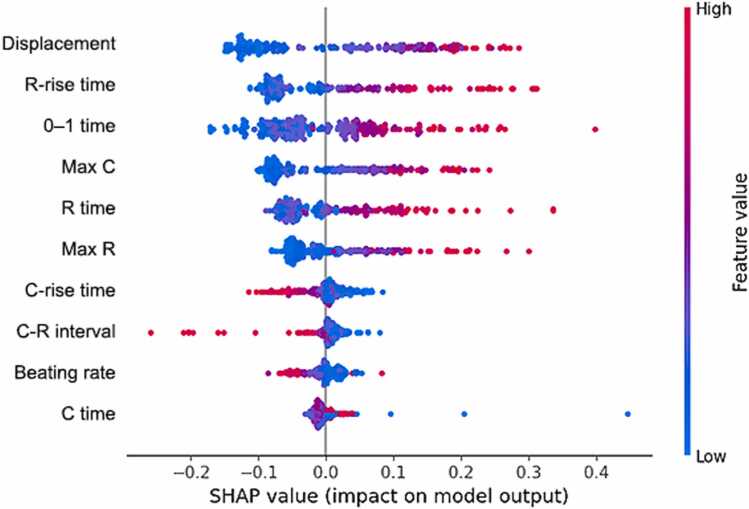
SHAP beeswarm plot of the features that were used to train the model as well as their influence on the classification outcome. Each dot represents one sample (i.e. one video). The position of the samples on the abscissa indicates the relevance of each feature for the classification of each sample as immature or mature iPSC-CMs. High positive or negative SHAP values indicate that the feature is very well suited to class separation for the corresponding sample. The color-coding of the samples indicates the magnitude of corresponding feature value, from blue for low feature values to red for high feature values. In the case of a perfect relationship between feature values and SHAP values, the coloring of the samples with increasing or decreasing SHAP values of the corresponding feature corresponds exactly to the color gradient of the color bar.

The distribution of the SHAP values of the respective features used as inputs are displayed in Fig. 5. The features were ranked by their impact on the classification outcome, depending on the feature values. Samples in which the corresponding feature had a high impact on the classification outcome were assigned with high SHAP values. Positive SHAP values indicate that the feature is relevant for the classification into a given class, whereas negative SHAP values indicate that the feature is relevant for rejecting a class. Large SHAP values indicate that the classification task is solved with high precision using a single feature. A SHAP value of 0 indicates that the feature is not relevant for classification. Outliers might increase the range but do not affect the impact ranking. Each sample/video is color-coded according to the magnitude of the corresponding feature value, thus illustrating the degree to which a given feature is expressed in that instance. The effectiveness with which a sample can be classified using a feature depends on the magnitude of the feature value. Consequently, a logical relationship is generally observed between the feature value and the SHAP value. In the context of a perfect relationship, a uniform color gradient is expected to form across samples of varying relevance.

The SHAP values of the feature displacement varied from –0.16–0.29, relaxation rise time from –0.13–0.31 and 0–1 time from –0.18–0.27 with an outlier at 0.41. Their ranges were 0.45, 0.44 and 0.45 respectively. The positive values were more stretched out, while the negative values were clustered closer together. The SHAP values of the Max C, the R time and the Max R showed a similar distribution. Max C ranging from –0.11–0.25, R time from –0.1–0.23 and Max R from –0.9–0.24. The SHAP value distribution of C-rise time, C-R interval and beating rate were clustered on the positive side, differing from the other features. C-rise-time ranges from –0.13–0.1, C-R-interval from –0.29–0.09 and beating rate from –0.09–0.07. The SHAP values of the C time were clustered around 0, ranging from –0.04–0.05.

Together, these results indicate that the first 6 features, displacement, R-rise time, 0–1 time, Max C, R time, and Max R, contributed most to the classification of a mature culture, with higher feature values supporting a positive classification, and lower feature values supporting a negative classification (Fig. 5). For these features, clustering occurred for samples with negative SHAP values, while samples with positive SHAP values were more widely scattered. This was consistent with parameter changes observed between immature and mature iPSC-CMs across individual cell lines (Supplementary Figure 2). In particular, high feature values of displacement and Max C matched positive SHAP values indicating a classification towards a mature culture, reflecting their robust increase in matured compared to immature iPSC-CMs across all 4 cell lines (Supplementary Figure 2 A, D). A correlation between high feature values and positive SHAP values was also observed for R-rise time, 0–1 time, R time, and Max R, although several samples with high feature values also showed negative SHAP values (Fig. 5). These results align with parameter increases in three of four lines for R-rise time, 0–1 time, and Max R (Supplementary Figure 2B, C, F), and in two of four lines for R time (Supplementary Figure 2E). In contrast, high feature values of C-rise time, C-R interval, and beating rate were associated with lower SHAP values, with lower feature values supporting the classification of a matured culture. However, the clustering for these features is less pronounced compared to displacement, R-rise time, 0–1 time, Max C, R time and Max R (Fig. 5). For C time, the SHAP values are very small and showed no clear relationship with feature values. Analysis of these features across the individual cell lines confirmed only small and inconsistent differences between immature and matured iPSC-CMs (Supplementary Figure 2 G-J), suggesting that changes in C-rise time, C-R interval, beating rate, and C time are less relevant for distinguishing immature from matured iPSC-CMs.

**Fig. 6:**
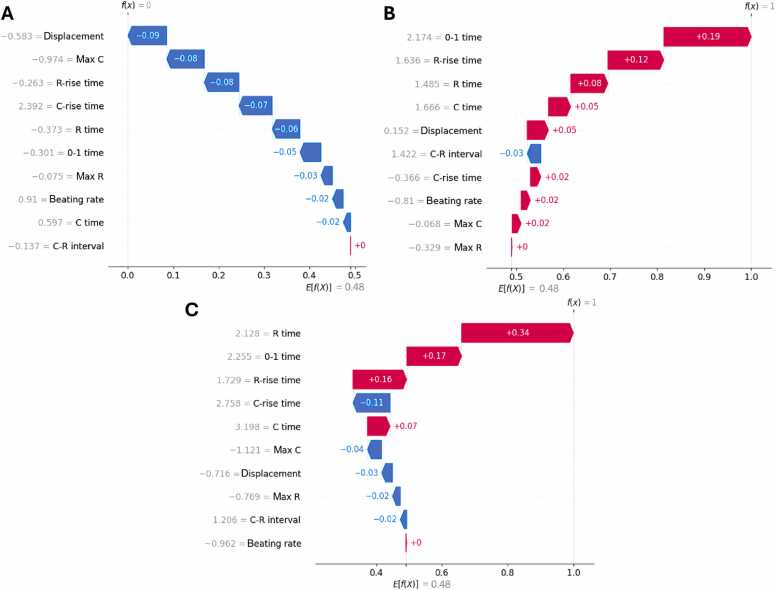
SHAP waterfall plots showing the feature importance for individual predictions of iPSC-CM cultures at different maturation states. A, Early state immature CMs at day 21. B, iPSC-CMs cultured in MM at day 42. C, iPSC-CMs cultured in B27 medium at day 42. The abscissa of the plots represents the expected classification outcome between immature (f(x)=0) and mature (f(x)=1). The contribution of the most relevant features to the classification result is shown line by line, from negative (blue) to positive (red) impact on the classification of an iPSC-CM as mature. The features are arranged in descending order of importance from top to bottom. The most relevant feature indicates the endpoint of the decision as either mature or immature. Each feature name is preceded by the corresponding normalized feature value.

Fig. 6 displays SHAP waterfall plots for a random representative cell culture of immature iPSC-CMs at day 21, mature iPSC-CMs cultured in MM medium at day 42 respectively, which were both classified correctly (Fig. 6A, B). The SHAP waterfall plots display explanations for individual predictions and thus allow insights into the individual feature importance for the classification of a sample as immature or mature iPSC-CMs. The 0–1 time and the features R-rise time and R time, correlating with the relaxation kinetics, showed the highest impact on the classification as matured state, whereas displacement and Max C had the strongest impact for classification into an immature state (Fig. 6A, B).

Next, we included video data from iPSC-CMs that were cultured in B27 medium from day 21 until day 42 (Fig. 6C). The iPSC-CMs cultured in B27 medium show a lower maturation state, as B27 medium mostly provides glucose as an energy substrate and only contains low levels of fatty acids [11]. Fig. 6C displays the SHAP waterfall plot for a random cell culture of iPSC-CMs cultured in B27 medium at day 42 that was classified as mature. In contrast to the examples presented in Fig. 6A and B, the features in this example are equally divided between the immaturity and the maturity of the iPSC-CM. While the magnitude of the C-rise time appears to contradictory to the maturity of the iPSC-CM; this is compensated by the contributions from R-rise time, 0–1 time, and R time (Fig. 6C).

**Fig. 7:**
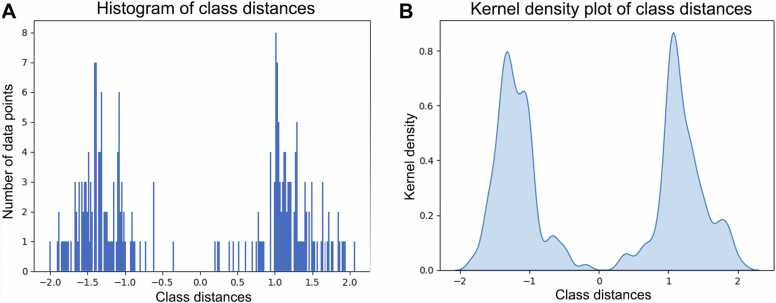
Visualization of classification into immature and mature cultures. A, Histogram of class distances illustrating the distribution of the distances of the samples from the hyperplane of the SVM. B, Kernel density plot showing the smoothed probability density of sample distances from the hyperplane SVM. The hyperplane is a separation plane in the feature space defined by the SVM to separate matured and immature cells. Samples with positive distances are classified mature, whereas samples with negative distances are classified as immature.

Furthermore, the decision function calculated the distance of each sample from the model's hyperplane, which reflects the separation plane for mature versus immature cultures. The 230 samples formed two gauss-like distributions (Fig. 7A). The left cluster represents the immature cell cultures (day 20/21 iPSC-CMs), whereas the right cluster corresponds to mature iPSC-CMs (day 42 in MM). The Kernel density plot shows a clear separation of the mature and immature samples, with no samples around the hyperplane at 0 (Fig. 7 B). Samples with the same distance from the hyperplane tend to have similar feature expressions and therefore a comparable maturity.

## 4 Discussion

Human iPSC-CMs are invaluable models for studying disease mechanisms, identifying novel drug targets and evaluating cardiotoxic or pro-arrhythmogenic substances [6], [8]. However, recent findings indicate that the drug responses of iPSC-CMs are strongly influenced by their maturation state. Consequently, assessing the maturity of iPSC-CMs may be beneficial to enhance reproducibility and reliability of experimental studies and drug screenings. Interestingly, recent studies have reported pronounced changes in the spontaneous beating behavior of less mature versus more mature iPSC-CMs [16], [17], [18]. These differences can be quantified using video-based motion analysis, a non-invasive method to characterize the contractile function of iPSC-CMs by measuring multiple features related to both contraction as well as relaxation [14], [28], [35], [36]. In this study, we aimed to investigate the potential of SVM models to assess the maturation state of iPSC-CMs based on beating features obtained via video-based motion analysis. Our analysis was based on beating data from iPSC-CMs with an improved maturation state through culture in lipid-supplemented MM (day 42) compared to less mature iPSC-CMs early after differentiation (day 20/21). Importantly, the enhanced maturation state of iPSC-CMs cultured in MM was previously confirmed through a variety of functional and molecular analyses [11].

Our results show that even with a relatively small data set of 230 videos, the SVM model is capable to discriminate between mature and immature iPSC-CM cultures with high accuracy (above 99 %). Good generalization is achieved, and the underlying characteristics of the maturity pattern are recognized with respect to the extracted features. To ensure the robustness of the model, cross-validation on the entire data set is recommended rather than relying solely on the hold-out test set. Using a small test set of 46 samples, the 5-fold cross-validation confirmed both the high accuracy and robustness of the model, with only one or two misclassifications observed in different folds. These results suggest that machine learning methods can be effectively applied to automatically assess the maturation state of iPSC-CMs using video-based beating features.

Investigation of the hyperplane of the SVM model, which represents the optimal data separation, revealed that some cultures that were classified as mature were close to the hyperplane. Further evaluation of the individual beating features of these iPSC-CM cultures indicated that this may be related to the individual iPSC-lines, likely reflecting their donor-specific genetic backgrounds. This suggests that, despite identical differentiation protocols and prolonged culture in MM, the feature values/expression can vary depending on the iPSC-line. This is reflected by the different effect of MM on the change of specific beating features such as R-rise time, 0–1 time, Max R and R-time, across the individual cell lines (Supplementary Figure 2).

A key advantage of assessing iPSC-CM maturation using beating features obtained via video-based motion analysis cultures is that this non-invasive approach can be flexibly applied during cultivation. Therefore, the SVM-based determination of the cell state may provide insight into whether a similar maturation state is reached across different independent experiments, potentially enhancing reproducibility through better synchronization of cellular conditions.

**Fig. 8:**
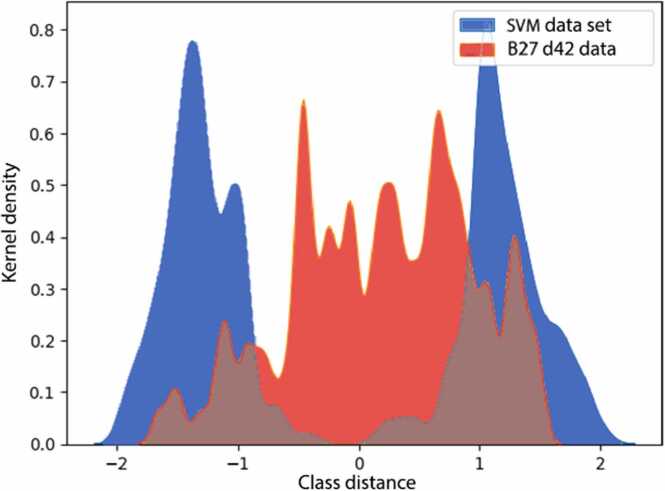
Kernel density function for immature iPSC-CMs on day 20/21 (left blue distribution), mature iPSC-CMs cultured in MM medium on day 42 (right blue distribution), and less mature iPSC-CMs cultured in B27 medium (red distribution). Blue colored distributions reflect the data set used for the training and validation process of the support vector machine (SVM).

When the SVM-model was applied to classify iPSC-CMs cultured in B27 medium, we found a heterogeneous distribution of these samples between the immature (day 20/21) and the mature cluster (MM at day 42), indicating a lower degree of maturation in comparison to iPSC-CMs cultured in MM (Fig. 8). This observation aligns with previous results demonstrating that MM promotes iPSC-CM maturation, including increased cell size, higher mitochondrial density, enhanced respiratory/metabolic activity, enhanced calcium handling and electrophysiological properties [11]. Therefore, these results suggest that the SVM model can distinguish multiple levels of maturity.

Next, we aimed to examine which beating feature or combination of features represent indicators to evaluate the maturation state of iPSC-CMs. The SHAP analysis revealed positive values for displacement, R-rise time, 0–1 time, Max C and R time, which demonstrates their importance for classifying matured cultures (Fig. 5). In contrast, C-rise time, C-R interval, beating rate, and contraction time tended to have negative SHAP values. The positive SHAP values of these features were concentrated in a narrow range, with a mean value below 0.1, and the contraction time exhibits outliers, indicating that these features had a limited impact on the model's decision-making process to classify immature and mature iPSC-CMs. Therefore, we identified that displacement, R-rise time, 0–1 time, Max C and R time were most relevant for maturity classification of iPSC-CMs. However, future studies are required to clarify how these beating features correlate with the molecular cellular properties associated with iPSC-CM maturation, particularly calcium handling and electrophysiology.

Previous studies have shown that the improved calcium handling of iPSC-CMs during maturation in fatty-acid supplemented media is reflected by reduced diastolic calcium levels [4], [11], increased transient amplitude [11], [37], [38] faster upstroke and decay kinetics [4], [11], [37], [38]. We hypothesize that the enhanced calcium upstroke and decay kinetics observed in iPSC-CMs cultured in MM [11] may be related to R-time, C-rise time, C-time and R-rise time, respectively [35] (Supplementary Figure 2). In addition, calcium transient duration may correlate with the 0–1 time, as indicated by previous studies which reported shortened calcium transient durations along with shortened contraction duration (measured via impedance) [39], [40]. Furthermore, linear correlations were reported between Max C (maximum contraction velocity) and contraction amplitude [36], as well as between displacement and traction force [40].

In addition to changes in calcium handling, iPSC-CMs cultured in MM showed remarkable differences with respect to their action potential properties and ion currents when compared to cells in B27 medium [11]. Hayakawa et al. simultaneously measured field potentials and motion in iPSC-CMs and observed strong correlations between field potential duration (FPD) and contraction duration [40]. In comparison to iPSC-CMs in B27 medium, iPSC-CMs cultured in MM showed increased densities of the sodium current (I_Na_), the transient outward potassium current (I_to_) and the inward rectifying potassium current (I_K1_); whereas the L-type calcium current (I_CaL_) was reduced [11]. These findings align with previous studies showing altered ion channel expression levels and current densities in iPSC-CMs compared to adult CMs. Adult CMs showed increased expression level/density of the sodium channel SCN5A/I_Na_, the hERG channel KCNH2/I_Kr_, and the potassium channel KCNQ/I_K1_, while the expression/density of the L-type calcium channel CANA1C/I_CaL_ was lower compared to iPSC-CMs [41]. These electrophysiological differences between adult CMs and immature iPSC-CMs may contribute to the discrepancies that were observed for the prediction of cardiotoxic or pre-arrhythmic activity using iPSC-CMs in comparison to the actual risk profile observed in the clinic [10], [41].

### 4.1 Limitations

Although video-based motion analysis can be easily established in laboratories to measure the contractile function of iPSC-CMs, the absolute values of beating features may be influenced by the use of different microscopy setups, cameras or analysis parameters (block width, max shift, frame offset). Therefore, it is crucial that the video data from iPSC-CMs intended for classification using our model match the technical specifications of our training dataset (0.65 µm/pixel, 60 FPS). Importantly, analyzing videos from iPSC-CMs with higher spontaneous beating frequencies, such as atrial-like iPSC-CMs, may require video acquisition at higher frame rates (100–150 FPS). Nevertheless, our study demonstrates that even a small dataset is sufficient to establish an AI-based model capable of distinguishing between immature and more mature iPSC-CMs based on their beating features. In our study, iPSC-CMs cultured in MM were assumed to be mature on day 42, but their maturation state is still very limited compared to adult CMs. Nevertheless, fatty acid-supplementation of the culture medium represents a simple and cost-efficient approach, without requiring specific materials, bioprinting or tissue engineering, and has been shown to improve electrophysiological development and drug response of iPSC-CMs [11]. Similarly, video-based motion analysis offers a simple, non-invasive and cost-effective approach to assess the contractile activity of iPSC-CMs. Therefore, we believe that SVM models like ours can be widely applied in labs working with iPSC-CMs. The video data used to train the SVM were obtained from iPSC-CMs derived from iPSCs lines of 4 healthy donors. Although robust classification was observed for all cell lines, it remains unclear whether the SVM is capable of correctly classifying iPSC-CMs derived from other iPSC lines with distinct genetic background. Future studies will focus on extracting beating features and classifying iPSC-CM maturity directly using the video data in order to incorporate for further features that have not yet been considered in our current model, such as the direction of motion or degree of alignment of the motion vectors.

## 5 Conclusion

In this paper, we implemented a SVM model that effectively distinguishes between immature early state (day 20/21) and matured (day 42) iPSC-CM cultures based on their beating features. The model achieved an accuracy of 99.5 % on a hold-out test set, demonstrating highly accurate classification [42]. The SHAP explanations reveal displacement, R-rise time, 0–1 time, and Max C to be the most impactful beating characteristics for discriminating immature and mature iPSC-CMs. Furthermore, application of the SVM model to classify iPSC-CMs cultured in B27 medium revealed their lower maturation state compared to iPSC-CMs in MM, consistent with our previous findings using various experimental techniques [11]. Therefore, this non-invasive approach allows the evaluation iPSC-CM maturity prior to drug testing or complex molecular and functional readouts, potentially improving reproducibility across independent experiments and accounting for variation between iPSC-CMs derived from different donors.

## CRediT authorship contribution statement

**Fabian Scheurer:** Writing – review & editing, Writing – original draft, Visualization, Validation, Software, Methodology, Investigation, Formal analysis. **Alexander Hammer:** Writing – review & editing, Writing – original draft, Validation, Supervision, Software, Project administration, Methodology, Investigation, Formal analysis. **Mario Schubert:** Writing – review & editing, Writing – original draft, Visualization, Supervision, Project administration, Methodology, Investigation, Formal analysis, Conceptualization. **Robert-Patrick Steiner:** Investigation, Formal analysis, Data curation. **Oliver Gamm:** Investigation, Formal analysis, Data curation. **Kaomei Guan:** Writing – review & editing, Supervision, Resources, Project administration, Methodology, Investigation, Conceptualization. **Frank Sonntag:** Writing – review & editing, Project administration, Methodology, Investigation, Conceptualization. **Hagen Malberg:** Writing – review & editing, Supervision, Project administration, Methodology, Investigation, Conceptualization. **Martin Schmidt:** Writing – review & editing, Writing – original draft, Visualization, Validation, Supervision, Project administration, Methodology, Investigation, Formal analysis, Conceptualization.

## Declaration of Competing interest

The authors declare that they have no known competing financial interests or personal relationships that could have appeared to influence the work reported in this paper.

## Data Availability

The research data and parameters referenced in this study are documented in the referenced publications. Maia software is freely available on Github (https://github.com/QuoData/Maia). For further inquiries or access to the datasets, interested parties may contact the corresponding author, who will provide data upon reasonable request. Please provide a clear hypothesis and study protocol.
