# Supplementary material for: Non-invasive maturity assessment of iPSC-CMs based on optical maturity characteristics using interpretable AI
Source: Comput Struct Biotechnol J. 2025 Aug 22;27:3719–28. doi: 10.1016/j.csbj.2025.08.024 (PMC12409467; doi:10.1016/j.csbj.2025.08.024)
Supplement: Supplementary file 1 — Supplementary material [file mmc1.docx]

**Supplementary Material**

Supplementary Figure 1


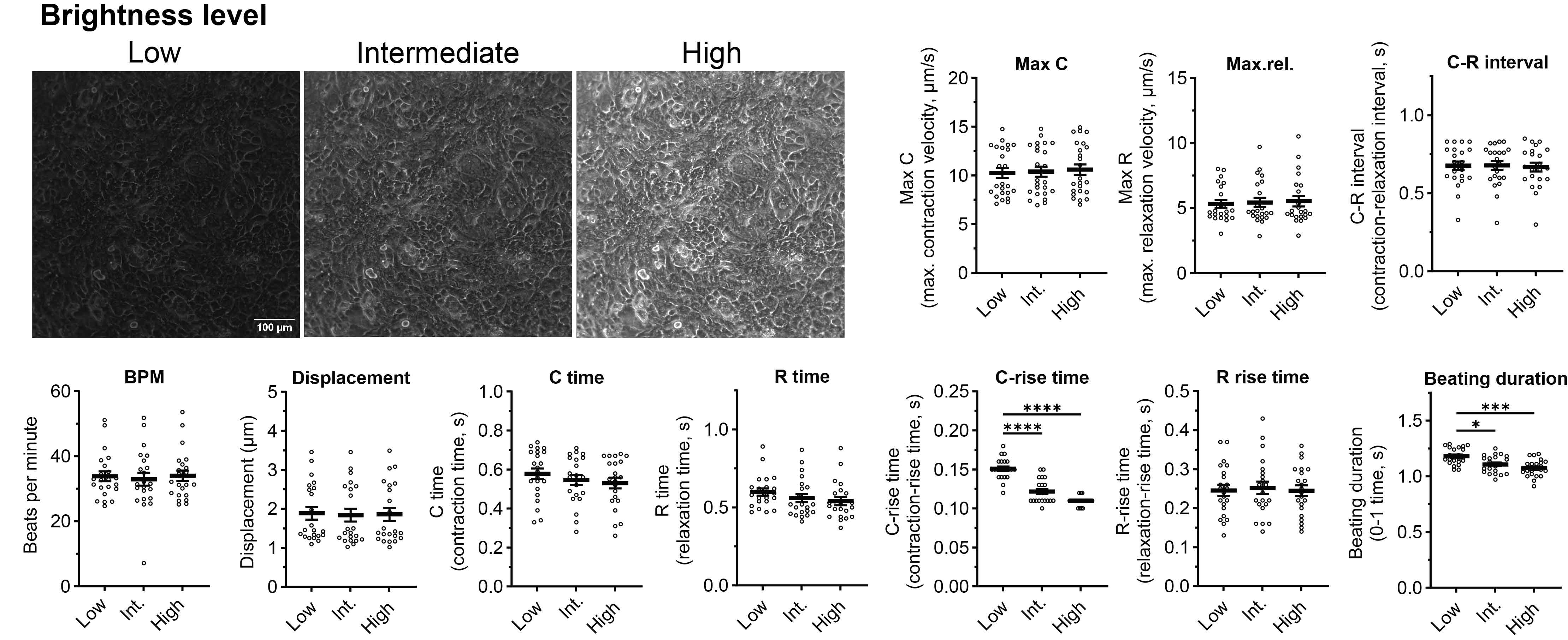


Supplementary figure 1: Influence of brightness levels on the individual beating parameters.Feature values extracted from videos (60 FPS, block width 10.4 µm, frame offset 67 ms (4 frames), max shift 4.55 µm) of an identical region of iPSC-CMs under different exposure/brightness levels. Data show mean and SEM from n = 22 videos/regions. Statistical analysis using Kruskal-Wallis test (*p < 0.05; ***p < 0.001; ****p < 0.0001).

Supplementary Figure 2


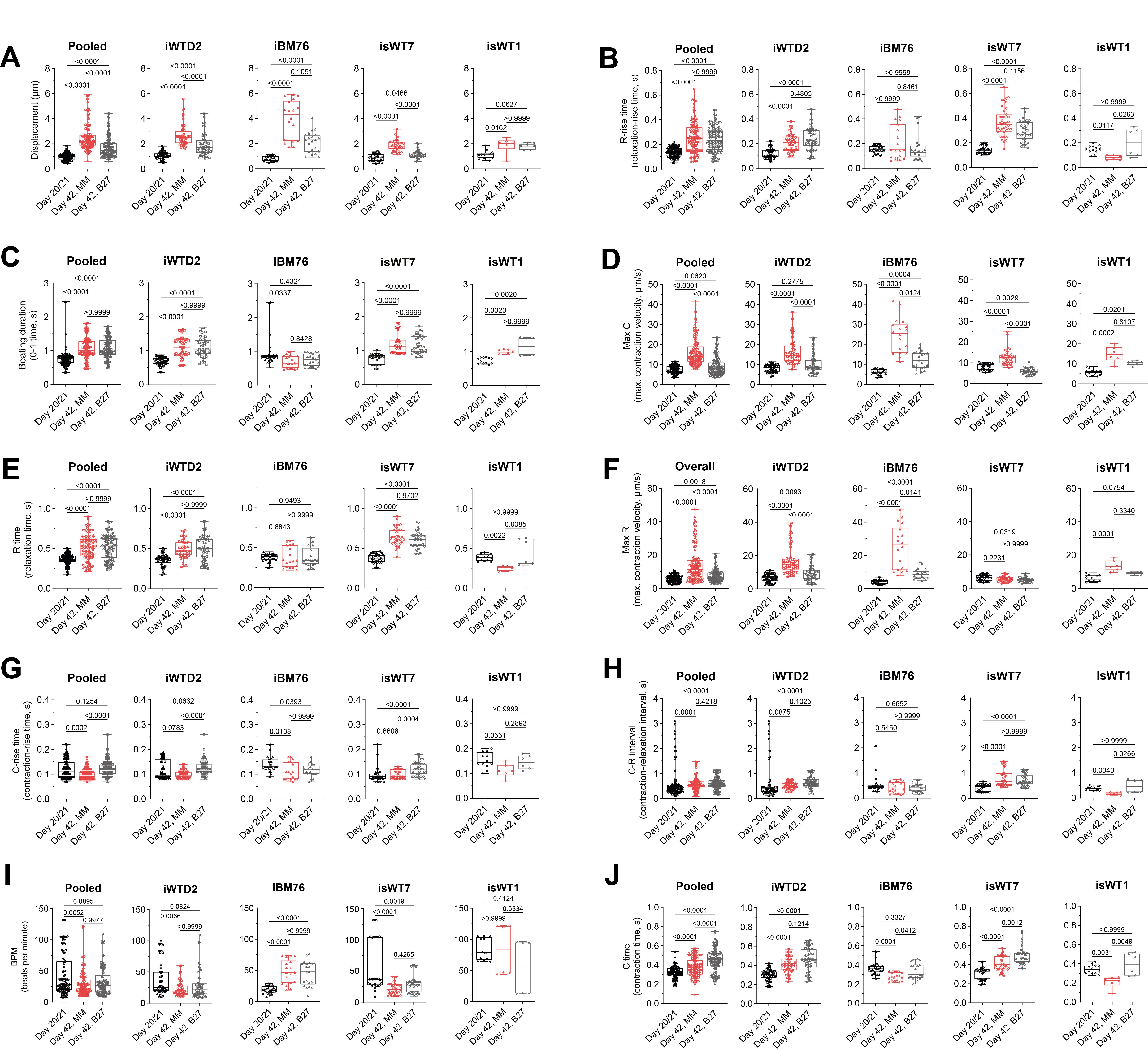


Supplementary figure 2: Beating characteristics of immature iPSC-CMs at day 20/21 and on day 42 after cultivation in MM (mature) or B27 medium (less mature). Box plots show pooled data (as depicted in Figure 4) and data of the individual cell lines. A-J; **A**, displacement; **B**, relaxation-rise time (R-rise time); **C**, beating duration; **D**, Maximum contraction velocity (Max C); **E**, relaxation time (R time); **F**, maximum relaxation velocity (Max R); **G**, contraction-rise time (C-rise time); **H**, contraction-relaxation interval (C-R interval); **I**, spontaneous beating rate; **J**, contraction time (C time). Data from n = 347 videos observed from 10 (day 20/21; n_21_ = 115), 16 (B27; n_42,B27_ = 117) or 14 (MM, n_42,MM_ = 115) independent differentiations of iPSC-CMs derived from iPSC-lines of 4 different donors (iWTD2, isWT7, iBM76, isWT1), as indicated by symbols. Statistical analysis was performed using Kruskal-Wallis test with Dunn’s correction for multiple comparisons.
